# Assessment of the efficacy of a fatigue management therapy in schizophrenia: study protocol for a randomized, controlled multi-centered study (ENERGY)

**DOI:** 10.1186/s13063-020-04606-6

**Published:** 2020-09-17

**Authors:** Stéphane Raffard, Nicolas Rainteau, Sophie Bayard, Yasmine Laraki, Joanna Norton, Delphine Capdevielle

**Affiliations:** 1 Univ Paul Valéry Montpellier 3, Univ. Montpellier, EPSYLON EA, Montpellier, France; 2 University Department of Adult Psychiatry, CHU Montpellier, Montpellier, France; 3 Inserm, Neuropsychiatry: Epidemiological and Clinical Research, Montpellier, France

**Keywords:** Fatigue, Schizophrenia, Negative symptoms, CBT, Sleep, Microbiota

## Abstract

**Background:**

Fatigue is a well-known common clinical feature of numerous chronic diseases including various forms of cancer, neurological disorders such as multiple sclerosis, and psychiatric disorders. A significant proportion of people with schizophrenia (30–60%) reportedly experience fatigue, which impacts negatively on participation in various activities, including work, study, leisure, and social pursuits. Causes of fatigue in schizophrenia are poorly understood and there are no established treatments. Several evidence-based interventions for fatigue syndrome including psychoeducation, cognitive behavioral therapy, and graded exercise therapy have been shown to be effective in other medical conditions and could be adapted to address fatigue in schizophrenia patients. As there are no psychosocial or pharmacological interventions with proved efficacy for fatigue in schizophrenia, there is an urgent need for the development of strategies to improve fatigue management in schizophrenia.

The aim of this project is to evaluate in a single blind randomized clinical trial the efficacy of a cognitive-behavioral therapy (CBT) intervention compared to treatment as usual (TAU) on fatigue as the main outcome in schizophrenia patients. Clinical symptoms, physical functioning, major cognitive functions, quality of life and functioning, treatment dosage, daily motor activity, biological markers with inflammatory markers are also considered as secondary outcomes.

**Methods/design:**

Two hundred patients meeting the inclusion criteria will be randomized to either of the study arms (intervention or TAU). The ENERGY intervention will be delivered according to a standardized treatment manual comprising six modules addressing fatigue and sleep over 14 individual therapy sessions. The treatment encompasses core CBT principles of psycho-education, behavioral activation, behavioral experiments, cognitive restructuring, problem-solving, and relapse prevention. Sessions will follow the traditional CBT structure of agenda setting, review of homework tasks, and introduction of a new concept/technique with collaborative discussions on how to implement such strategies in the participant’s day-to-day environment. Our primary endpoint will be the severity of fatigue assessed at baseline and at the 9-month follow-up using the “Multidimensional Fatigue Inventory” (MFI).

**Discussion:**

The trial will provide the first test of CBT intervention for fatigue for patients with schizophrenia. This study will also test to what extent the treatment can be implemented in everyday practice.

**Trial registration:**

ClinicalTrials.govNCT04332601. Registered on 10 April 2020.

## Introduction

### Background

Schizophrenia is probably the most debilitating of all the mental health disorders. Rates of schizophrenia are similar from country to country—affecting approximately 0.5 to 1% of the population [1]. People diagnosed with schizophrenia usually experience a combination of symptoms [2]: positive (i.e., hallucinations, delusions), negative (i.e., amotivation, reduced expressed emotions, poor or non-existent social functioning), cognitive (i.e., difficulty concentrating, memory impairments), affective (i.e., depression), and disorganization (i.e., disorganized speech, inappropriate affect). Even if medication is considered as the first-option treatment for positive symptoms and relapses in schizophrenia [3], the proportion of individuals who achieve a clinically meaningful benefit is moderate [4]. This can be partly explained by the fact that no pharmacological treatment has been shown effective in treating negative [5] or cognitive symptoms, which are the major cause of the marked functional disability associated with schizophrenia [6]. In addition, other non-psychiatric clinical conditions which are often not the primary focus of clinicians treating this population may have been overlooked [7].

To date, fatigue in patients with severe mental disorders such as schizophrenia has been poorly studied despite several arguments for further investigation [8]. First, lack of energy, one of the main characteristics of fatigue, is frequently encountered in individuals with schizophrenia. Second, fatigue is a common and severe symptom in sleep disorders [9] and depression [10] which both are commonly observed in individuals with schizophrenia [11]. Third, fatigue and psychiatric disorders frequently occur comorbidly, and fatigue may aggravate current existing psychiatric conditions [12].

#### Gap and research hypothesis

Fatigue is a subjective phenomenon defined as “the awareness of a decreased capacity for physical and/or mental activity due to an imbalance in the availability, utilization, and/or restoration of resources needed to perform activity” [13]. Fatigue is common in psychiatric conditions such as anxiety and depression (25–36%) as well as in chronic medical conditions such as cancer, Parkinson’s disease, and diabetes [14, 15]. Problems concerning fatigue are often overlooked due to their complexity [16]. The overlap in symptomatology between psychiatric disorders and fatigue can make it difficult to disentangle complex comorbidities [7, 12]. Indeed, fatigue in its different manifestations is sometimes perceived as a symptom and sometimes a condition mainly communicated by the affected person, with a severe impact on quality of life and interpersonal relations [14, 16, 17]. As a result, studies of fatigue in patients with psychosis are currently lacking. Yet, several arguments support the need to develop effective interventions for the treatment of fatigue symptoms in these patients. First, fatigue is highly prevalent in schizophrenia, with almost 60% of patients reporting clinically significant levels of fatigue [8]. Second the functional consequences of fatigue include considerable impairment and disability, such as amotivation to engage in physical activity [18], cognitive deficits, reduced social interactions, and poorer functional health [8], pointing to fatigue as an important treatment target. Third, fatigue may worsen current existing psychiatric conditions, particularly negative symptoms including apathy, decreased interest, or sustained attention, which poorly respond to pharmacological and psychological treatment.

#### Originality and innovative aspects of the study

No psychosocial or pharmacological interventions exist with proved efficacy on fatigue in schizophrenia. But several interventions including cognitive behavioral therapy (CBT), sleep hygiene education, and graded exercise therapy are effective in other chronic medical conditions such as stroke or chronic fatigue syndrome. There is an urgent need for the development of strategies to improve fatigue in schizophrenia. Another innovative aspect of our project is to develop an integrative model based on biological and psychological factors underlying fatigue severity in schizophrenia such as sleep disorder but also inflammatory processes and microbiota. Fatigue is common in individuals with a chronic illness with an overriding inflammatory process. Uncontrolled neuro-inflammation by pro-inflammatory cytokines is involved in the pathogenesis of schizophrenia. The intestinal microbiota plays a critical role in the immune response [19], with communication between the microbiota and gut mucosal cells regulating the production of cytokines and chemokines. A management of fatigue could decrease this inflammatory syndrome and thus the bacterial translocation.

### Objectives

#### Primary objective

The primary objective in this study is to assess in a randomized, single blind, multi-centered clinical trial the efficacy of an individual cognitive and behavioral therapy protocol versus TAU, on schizophrenia patients’ fatigue severity with a follow-up at 9 months after inclusion.

#### Secondary objectives

Secondary objectives are to examine change in fatigue score over three time points (inclusion to 3 months to 9 months), according to the study arms. Another objective is to assess at the 9-month follow-up the impact of the intervention on:

- Clinical symptoms (positive and negative symptoms, depressive symptoms, insight, sleep)
- Physical functioning
- Major cognitive functions (attention, memory and executive functions)
- Quality of life and functioning
- Treatment dosage
- Daily motor activity
- Biological markers (weight, abdominal perimeter, blood pressure, heart rate, lipids, inflammatory markers)

### Hypotheses

The primary hypothesis is that:

1. Compared to the control condition (TAU), ENERGY intervention will lead to a reduction in fatigue severity (at the posttreatment and 9-month follow-up)

The secondary hypotheses are that:

2. Compared to the control condition, ENERGY intervention will lead to reductions in negative symptoms and more particularly amotivation and depression severity and will lead to increases in daily motor activity, physical functioning, quality of life, and sleep (posttreatment, 9-month follow-up).

## Methods: participants, interventions, and outcomes

### Participants

Two hundred individuals with schizophrenia will be recruited in 9 centers. Each participating center will include 20 eligible participants in the study, except for the Montpellier center which will include 40 participants. The study protocol has been approved by the Ethical Committee (CNRIPH: 19.11.29.43226) in accordance with the Helsinki Declaration. Written informed consent will be obtained by all participants. The study report will comply with the Consolidated Standards of Reporting Trials (CONSORT) statement and CONSORT-NPT (non-pharmacologic treatments) statement.

### Eligibility criteria

#### Inclusion criteria

Patients eligible in participating in this study must comply with the following criteria:

1. Age between 18 and 60 years.
2. Signature of informed consent form. If patients have a tutor, they will have to sign the informed consent form too.
3. Patients with a diagnosis of schizophrenia according to the Diagnostic and Statistical Manual of Mental Disorders 5 (DSM 5) established and validated by the study center’s investigating psychiatrist.
4. Patients with a score at the “Multidimensional Fatigue Inventory” (MFI) greater or equal to 10.
5. Patients with a follow-up in a day hospital at inclusion.
6. Subjects must be able to attend all scheduled visits and to comply with all trial procedures.
7. Participation of the patient must be noted in their medical files.
8. Subjects must be covered by public health insurance.

#### Exclusion inclusion criteria are

Patients will not participate in this study if they meet any of the following criteria:

1. Subject unable to read and/or write French
2. Planned long-term stay outside of the study region that prevents compliance with the treatment plan
3. Patients with a history of severe brain trauma
4. Patients with a history of neurological pathology
5. Pregnant or breastfeeding patients

### Study design and setting

This ENERGY trial is a two-arm study (ENERGY intervention versus TAU), single blind randomized and controlled clinical trial designed to assess the efficacy of an individual cognitive and behavioral therapy in patients with schizophrenia. We adhered to the Consolidated Standards of Reporting Trials (CONSORT) guidelines in the design of the trial.

Patients meeting all of the inclusion criteria and none of the exclusion criteria will be invited to participate in the study. After signing the informed consent form, they will be randomized into one of the two study arms: ENERGY intervention or TAU.

They will be assessed at baseline over a 1-week period for fatigue, global functioning, symptomatology, sleep, cognitive functioning, and antipsychotic medication. Blood samples will be collected. Patients will be re-assessed 3 months later (corresponding approximately to the end of the ENERGY intervention; if the intervention is delayed, this intermediate assessment will be performed as soon as the intervention is completed and within 15 days of the end of the intervention) and 9 months later (approximately 6 months after the intervention with a delay of 15 days maximum) for fatigue, symptomatology, global functioning, sleep, cognitive functioning, and antipsychotic medication. Blood samples will be collected at each of these assessment times.

This study is multi-centered and will be carried out in 9 of the 10 centers participating in the French national network of schizophrenia expert centers (FondaMental Foundation). Each center will recruit patients to both groups and therefore perform the ENERGY intervention. The following centers will be participating in this study:

1. Hôpital la Colombière, Centre Hospitalier Universitaire (CHU) de Montpellier
2. CHU de Clermont Ferrand
3. Hôpital Albert Chenevier, Créteil
4. Hôpitaux Universitaires de Strasbourg
5. Hôpital Sainte Marguerite, Marseille
6. Hôpital Louis Mourier, Colombes
7. Hôpital le Vinatier, Lyon
8. Centre Hospitalier Alpes Isère
9. Hôpital Charles Perrens Bordeaux

This study protocol has been reported in accordance with the “SPIRIT 2013 Checklist: Recommended items to address in a clinical trial protocol and related documents” (Additional file 1).

### Justification of the study design

This clinical trial is designed to assess the efficacy of an individual cognitive and behavioral therapy in patients with schizophrenia by comparing results from the ENERGY intervention to TAU.

We chose a single blind study to ensure that the psychologist performing the evaluations will be blinded to the randomization arm. It is not possible for patients following the ENERGY intervention to be in a blinded condition.

For the comparison group, called “treatment as usual” (TAU), no definition of classical usual care for patients with schizophrenia exists; the only consensus is that antipsychotic medication is essential [20, 21]. Furthermore, French data show a high rate of care in day hospitals for patients suffering from schizophrenia, with day hospital care accounting for 40% of outpatient care [22]; 84% of French sectors have at least 1 day hospital [23]. So, the TAU could be defined by antipsychotic medication coupled with day hospital care.

### Intervention description

#### Experimental group

- Treatment as usual (see below)
- ENERGY intervention:
  - Fourteen sessions of 1-h CBT intervention
  - One session per week
  - With a psychologist (different to the psychologist who will perform the assessments)
  - Individual sessions

ENERGY intervention is a cognitive-behavioral intervention developed by Knoop and Bleijenberg [24] and adapted in French by Cantin and colleagues [25]. The ENERGY intervention will be delivered according to a standardized treatment manual comprising six modules addressing fatigue and sleep over 14 individual therapy sessions (Table 1). The treatment encompasses the core CBT principles of psycho-education, behavioral activation, behavioral experiments, cognitive restructuring, problem-solving, and relapse prevention [26]. Sessions will follow the traditional CBT structure of agenda setting, review of homework tasks, and introduction of a new concept/technique with collaborative discussion on how to implement such strategies in the participant’s day-to-day environment [26].

**Table 1:** Treatment modules and therapeutic aims

Module	Content summary	Aims
1	Psychoeducation CBT Framework	Shift unhelpful beliefs around symptoms Encourage understanding of link between activity levels and sleep habits with syndrome maintenance
2	Reorganize daily schedules	Initiate pacing of tasks, with optimal number of rest breaks Increase hygiene practices
3	Energy conservation	Integrate meaningful activities depending on goals and available energy
4	Cognitive restructuring Sleep interventions	Modify rest and sleep appraisals Stimulus control, bedtime restriction, hypersomnia management Relaxation
5	Strategies for physical and mental fatigue	Environmental modification, restructuring tasks Cognitive strategies for information processing, attention and time pressure management
6	Review techniques Relapse prevention	Consolidate treatment gains

The CBT framework for addressing fatigue and sleep disturbance was adapted from previous treatment manuals for Chronic Fatigue Syndrome and insomnia, with further modifications made to its content and delivery [27, 28]. To account for possible cognitive impairments frequently encountered in schizophrenia, a specific focus was made on implementing cognitive strategies (e.g., preventing information overload, memory aids, time-pressure management), specific napping schedules, and re-organizing activity levels as a means of energy conservation in addition to pacing and graded activity exposure. Deficits in memory and executive functioning are common in individuals with schizophrenia and may be further exacerbated by poor sleep and persisting fatigue. Relative to conventional CBT, greater structure was imposed in sessions with concrete principles as well as more directive guidance from therapists in problem-solving and generating alternative helpful thoughts. Simplified hand-outs with pictorial cues were provided after each session to minimize information overload and demands on memory. External aids such as diaries and reminders were used to prompt participants to complete prescribed tasks across the week.

#### Control group

Treatment as usual comprises antipsychotic medication, day hospital, and ambulatory care during all the study.

Concomitant care: no concomitant care is prohibited during the trial.

### Outcome measures

We will use a majority of French validated scales and cognitive tests, designed for and commonly used in schizophrenia patients.

#### Primary outcome measures

The primary outcome measure is the score at the Multidimensional Fatigue Inventory (MFI) [29]. The MFI is a self-assessment instrument with 20 items including 5 dimensions: General, Physical, and Mental Fatigue; Reduced Motivation; and Reduced Activity. It was validated in 93 patients with schizophrenia spectrum disorders. The test–retest showed a correlation between .66 and .91 for all subscales of MFI. The internal consistency was .92. The analysis of convergent construct validity showed a correlation of .68 (time 1) and .77 (time 2). No item was systematically identified as being difficult to answer [30].

#### Secondary outcomes measures

Secondary outcome measures will include global functioning, symptomatology, sleep, cognitive functioning, and antipsychotic medication, as well as blood samples:

##### Symptomatology

Positive and Negative Syndrome Scale (PANSS) [31]: The PANSS is a 30-item scale designed to note specific symptomatology of psychotic disorders from 1 to 7. The results include a positive, negative, general psychopathology, and total score allowing categorical and dimensional quotation. The 5 dimensions of this scale were validated in French [32].

Clinical Assessment Interview for Negative symptoms (CAINS) [33]: The Clinical Assessment Interview for Negative Symptoms (CAINS) was developed through a multistage scale development process to measure current level of severity of negative symptoms in individuals with schizophrenia and schizoaffective disorder. The time frame for the ratings is the previous week. The scale was designed for use in treatment trials, but it can also be used in other types of negative symptom research.

The CAINS comprises two subscales: the nine-item Motivation and Pleasure scale and the four-item Expression scale. A composite of the two subscales can be computed.

All ratings are based on a semi-structured interview with prompts and queries. Items are rated on a 5-point (0–4) scale, ranging from 0 (absence of symptom) to 4 (severe symptom level). It should be noted that lower “impaired” scores on several of the items might be within the range of normal variation in the general population. All ratings should be made based solely on descriptors in the anchors without attempting to compare them to clinical or non-clinical reference groups. French validation is in progress by our team with author permission.

Calgary Depression Scale for Schizophrenia (CDSS) [34]: the CDSS is an easy and quick 9-items scale, designed specifically to evaluate depression in schizophrenic patients. The scale was derived from two existing scales by factor analysis and reliability analysis. It has been further tested in two samples. In the first, it has been shown to be reliable, congruent with a self-report scale, and valid. In the second sample, it has been shown that there is no overlap with negative or extrapyramidal symptoms [34]. THE CDSS has been validated in French [35].

##### Sleep

Berlin Questionnaire (BQ) [36]: The questionnaire consists of 3 categories related to the risk of having sleep apnea. The BQ incorporates questions about snoring (section 1), daytime somnolence (section 2), and hypertension and BMI (section 3). In the first section, the participants are asked to score their snoring. In the second section, daytime fatigue and sleepiness during daily activities are investigated, and in the last section, medical history, demographic, and anthropometric measures, such as height and weight, can be evaluated. The first two sections are assumed to be positive, if the total score is 2 or more. If a patient has hypertension or a BMI higher than 30 kg/m^2^, section 3 can be considered positive. In general, if there are two or more sections with positive scores, this subject is categorized as “high risk” for OSA (obstructive sleep apnea).

Idiopathic Hypersomnia Severity Scale (IHSS) [37]: The IHSS is a self-report measure of hypersomnolence symptoms, consequences, and responsiveness to treatment. The IHSS is a 14-item self-administered questionnaire that assesses nighttime sleep symptoms and the related sleep inertia (5 items), daytime sleep symptoms and the related sleep inertia (4 items), and impaired daytime functioning due to hypersomnolence (5 items). All items are scored from 0 to 3 or 4, depending on severity, that provide a score, ranging from 0 to 50, with the highest score reflecting more severe and frequent symptoms.

Sleep Condition Indicator (SCI) [38]: The SCI is a brief 8-item tool to evaluate insomnia based on DSM 5 criteria. The SCI assesses night time and daytime symptoms and focuses both on the frequency and persistency of these symptoms. The French version of the Sleep Condition Indicator demonstrates satisfactory psychometric properties while being a useful instrument in detecting cases of insomnia disorder, consistent with features of DSM-5 [39].

##### Physical functioning

International Physical Activity Questionnaire short form (IPAQ) [40]: The IPAQ considers a 7-day recall period, identifying physical activity undertaken in the morning, afternoon, and evening. For the purposes of this research, the questionnaire was interviewer administered. Data from the IPAQ is summarized according to minutes per week of walking, minutes per week of moderate physical activity (e.g., activities that take moderate physical effort and make you breathe somewhat harder than normal such as carrying light loads, bicycling at a regular pace, or easy swimming), and minutes per week of vigorous physical activity (e.g., activities that take hard physical effort and make you breathe much harder than normal such as heavy lifting, digging, aerobics, or fast bicycling) per week. Previous research demonstrated that the IPAQ is a reliable surveillance tool to assess levels of physical activity in patients with schizophrenia [41].

##### Quality of life and general functioning

Quality of life (S-QoL) [42]: The S-QoL is a self-administered instrument to assess health-related quality of life (HRQL) among people with schizophrenia. The S-QoL, based on Calman’s approach to the subject’s point of view, is a multidimensional instrument that is sensitive to change. The scale is a 41-item questionnaire with eight subscales (psychological well-being, self-esteem, family relationships, relationships with friends, resilience, physical well-being, autonomy, and sentimental life) and a total score. The validation study showed high internal consistency reliability, reproducibility, and responsiveness. Construct validity was confirmed using established clinical and HRQL measures. The S-QoL is an efficient instrument for the measurement of the impact of schizophrenia on individuals’ lives.

Functional Remission of General Schizophrenia (FROGS) [43]: The FROGS evaluates patient functioning. It was developed using the expert consensus method following a MEDLINE and standard database search. Nineteen items were selected as gathering the core aspects of functional remission in schizophrenia detected in the literature. The FROGS was then evaluated in 432 patients with DSM-IV criteria of schizophrenia, all of them meeting the symptomatic remission criteria, total scores were shown to be highly reliable. Exploratory factor analysis after oblique rotation revealed that a three-factor solution was the most meaningful. On the basis of item content, these three factors were labeled “Social Functioning,” “Daily Life,” and “Treatment.” The FROGS total score can be used to measure a general construct for the evaluation of functional remission in schizophrenia.

Intrinsic Motivation Inventory for Schizophrenia Research (IMI-SR) [44]: Intrinsic motivation is an important mediating factor between neurocognition and psychosocial outcome. The IMI-SR is a self-report IM scale that gauges the central motivational structures identified by Self-Determinism Theory as pertinent to cognitive task engagement, skill acquisition, treatment compliance, and remediation outcome. The IMI-SR is a concise instrument, possessing good internal consistency and test-retest reliability. The final form has 21-item questionnaire comprised of 3 domains relevant to motivation for treatments (interest/enjoyment, perceived choice, value/usefulness). The IMI-SR is a viable instrument to measure intrinsic motivation in schizophrenia as part of a cognitive remediation protocol or psychosocial rehabilitation program.

All the previously mentioned measures will be implemented at baseline, 3-month, and 9-month follow-up.

##### Cognitive assessment

French National Adult Reading Test (fNART) [45]: The test consists of 40 words, graded in difficulty, whose pronunciation cannot be determined from their spelling. The ability to successfully read irregularly spelt words is relatively robust in the face of current cognitive impairment and is a sensitive marker of intellectual attainment. The fNART is a reliable and valid method of assessing premorbid intellectual ability in French speakers.

California Verbal Learning Test (CVLT) [46]: The CVLT was developed to evaluate in a multifactorial way verbal learning and memory. It has the particularity to quantify several cognitive components of verbal memory in only one test. Its duration is around 20 min. A list of 16 words (four from each of four semantic groups) is presented orally to the participants, who are required to recall the words immediately, after short and long delays and also with and without semantic cues. A recognition task is then administered. The test includes the following measures to reflect learning and recall, also called primary variables: Total Trial 1–5, Short Delay Free Recall (SDFR), Short Delay Cued Recall (SDCR), Long Delay Free Recall (LDFR), and Long Delay Cued Recall (LDCR). It also includes other variables that reflect processing of information and other cognitive processes such as Semantic Clustering and Serial Clustering, Region effects, Intrusions, and Perseverations.

Trail Making Test (TMT) [47]: Trail Making Test reflects the control of attention, visual exploration, speed, and mental flexibility. The subject is asked to connect, by making pencil lines, encircle numbers randomly arranged on a page in proper order (Part A) and encircle numbers and letters in alternating order (Part B). A French version of the normative data will be used.

##### Biological measures

The following biological measures will be taken at baseline, 3-month, and 9-month follow-up: weight, BMI, abdominal perimeter, hearth rate, and blood pressure.

Pedometer: is an electronic portable device, which counts each step a person takes by detecting the motion of the person’s hands or hips. Distance traveled (by walking or any other means) can be measured directly by a global positioning system (GPS) receiver. Our objective is to measure steps and distance covered by patients. Each patient will wear the pedometer during 1 week, 3 times during the trial: at inclusion, at 3 months just after the end of the therapy, and at 9 months just before the end of the study.

- Glycemia, lipids, and C-reactive protein (CRP) will be performed with only one tube of 5 ml (dry tube)
- Granulocyte-macrophage colony-stimulating factor (GM-CSF), interferons (IFN)-γ, interleukins (IL)-1β, IL-2, IL-4, IL-5, IL-6, IL-8, IL-10, and tumor necrosis factors (TNF)-α: This kit is comprised of components for the measurement of human GM-CSF, IFN-γ, IL-1β, IL-2, IL-4, IL-5, IL-6, IL-8, IL-10, and TNF-α in serum, plasma, or tissue culture supernatant. They will be tested by enzyme-linked immunosorbent assays: Commercially available enzyme-linked immunosorbent assay (ELISA) kits will be used according to the manufacturers’ recommendations for measuring concentrations of intestinal fatty acid binding protein (I-FABP) (Clinisciences), soluble cluster of differentiation 14 (CD14) (Enzo), lipopolysaccharide binding protein (LBP) (Enzo), and zonulin (Servbio).

### Microbial translocation

#### DNA extraction

Plasma samples will be collected in Ethylenediaminetetraacetic acid (EDTA)-anticoagulated tubes and stored at − 80 °C. Deoxyribonucleic acid (DNA) will be extracted from 200 μL of plasma using the EZ1 DNA Tissue kit (Qiagen, Courtaboeuf, France) according to the manufacturer’s instructions. DNA will be eluted in a 100-μL final volume.

#### 16S rDNA real-time PCR

Bacterial 16S rDNA levels will be measured by polymerase chain reaction (PCR). A 20-μL amplification reaction consisted of 4 μL of LightCycler FastStart DNA Master^PLUS^ HybProbe (Roche, Meylan, France), 0.5 μmol/L forward and reverse primers, 1 μmol/L TaqMan probe, and 10 μL of the template plasma DNA. Degenerate forward (16S-F: 5′-AACAGGATTAGATACCCTGGTAG-3′ nucleotide 780 to 802) and reverse (16S-R: 5′-GGTTCTKCGCGTTGCWTC-3′ nucleotide 962 to 979) primers will be used to amplify the hypervariable region V5 of the 16S gene. Carboxyfluorescein stained probe (16S-probe: 5′-fluorescein amidites (FAM)-AACAC5TGCTCCACCGCT-BHQ1-3′ nucleotide 937 to 948, where FAM means 6-carboxyfluorescein and BHQ1 means Black Hole Quencher I) will be used as described by Kramski and colleagues [48]. The amplified region is 199 bp. The DNA is independently amplified twice in duplicate, and mean values are calculated. A negative control (molecular biology grade water) is systematically used. A standard curve is created from serial dilutions of plasmid DNA containing known copy numbers of the template. The reaction conditions for amplification of DNA are 95 °C for 10 min, followed by 45 cycles at 95 °C for 15 s, 60 °C for 60 s. The assays are performed using a LightCycler 480 II (Roche), with absolute quantification analysis performed with the Lightcycler 480 software (Roche), version 1.5, according to the manufacturer’s recommendations.

### Other data collected

#### Socio-demographic data

At inclusion, we will record age, gender, education level, marital status, occupation, living status, and income level.

#### Medical history

At inclusion, we will record age at first episode, duration of untreated psychosis, comorbid disorder including addictions, and other somatic diseases.

Current drug therapy and psychosocial interventions will be recorded at each visit.

### Biological sampling collection

We will collect blood samples:

- Five milliliters on dry tube for glycemia, CRP, and lipids on dry tube
- 1.5 ml for microbial translocation on dry tube
- Five milliliters for interleukins and cytokines on dry tube

Glycemia, CRP, and lipids will be dosed in each of the 10 centers and results noted in the eCRF.

Microbial translocation analysis will be centralized in Nîmes, U1047 Inserm Unit, and interleukins and cytokines analysis will be centralized in CHU Montpellier.

Samples will be stored in each center in a − 80 °C fridge until the end of the study and sent to the analysis centers with dry-ice.

The blood samples for cytokines need to be centrifuged before freezing within 24 h from time of collection. Samples will be stored in Montpellier and preserved for 15 years.

### Participant timeline

**Fig. 1:**
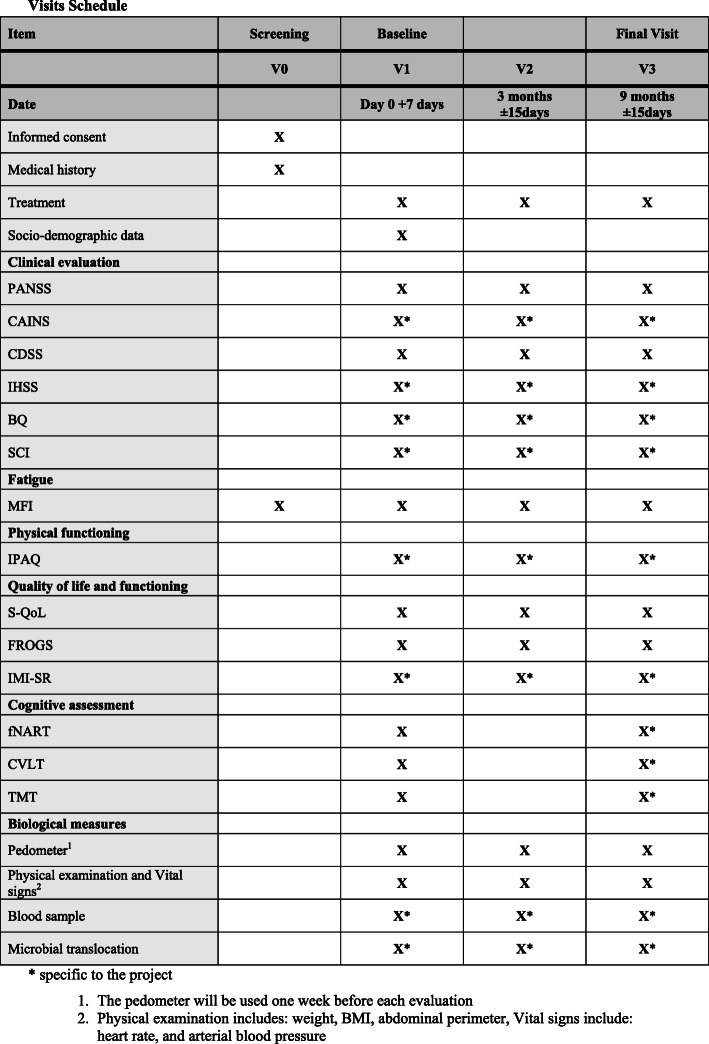
Visit schedule

Participants will be assessed at the beginning of the study, then re-assessed 3 months later (corresponding approximately to the end of the ENERGY intervention; if the intervention is delayed, this intermediate assessment will be performed as soon as the intervention is completed and within 15 days of the end of the intervention) and 9 months later (approximately 6 months after the intervention with a delay of 15 days maximum) for fatigue, symptomatology, global functioning, sleep, cognitive functioning, and antipsychotic medication. Blood samples will be collected at each of these assessment times (see Fig. 1).

### Planned trial calendar

Planned regulatory period: 6 months

Planned duration of recruitment: 36 months

Duration of participation of each subject: 9 months

Planned statistical analysis/valorization period: 6 months

Planned total trial period: 57 months

### Determination of sample size and power calculation

The main objective is to evaluate the efficacy of a therapeutic protocol for the management of fatigue in schizophrenia patients. The change in fatigue level will be compared 6 months after the intervention (9-month follow-up) between two groups of patients: one following the 3-month individual program for the management of fatigue, and one receiving care as usual. The sample size calculation is based on the change in the general fatigue score, one of the five sub-scales of the Multidimensional Fatigue Inventory (MFI) [29], measured at inclusion and 9 months later.

A study exploring the psychometric properties of the MFI in 93 out-patients with schizophrenia spectrum disorders reported a mean score on the general fatigue subscale of 13.4 (standard deviation (SD): 3.9) [30]. An earlier study reported a mean score of 16 (SD 3.2) for this sub-scale in 166 patients with fibromyalgia and chronic widespread pain [49].

In our study of SZ patients attending a day hospital, with a general fatigue subscale score greater or equal to 10 at study entry, we can expect an average score of approximately 16 (SD 4) on this subscale.

We can also hypothesize a 2-point reduction in the mean score on this sub-scale between the start of the trial and the 9-month follow-up in the group receiving the intervention. No change in the severity of fatigue is expected in the group receiving treatment as usual. Under this effect-size hypothesis, with scores in both groups set at 16 (SD 4) at inclusion, the mean score in the group receiving the intervention is expected to drop to 14. Under the hypothesis of a stable standard deviation of 4 in both groups and at both time points, power set at .085 and alpha at *p* = 0.05, 73 patients per group are necessary.

In order to allow for loses at follow-up (estimated for SZ patients at 15–20% over a 9-month period), it will be necessary to recruit 100 subjects per group.

The 9 centers participating in this study are part of the French network of expert centers in schizophrenia of the FondaMental foundation. Participants will be recruited directly from the day hospitals in each of the centers.

## Methods: assignment of interventions

### Randomization and matching

After checking for all inclusion and non-inclusion criteria by the study investigator, patients will be randomized to either of the study arms (intervention or TAU). Allocation concealment will be guaranteed as the randomization will not be released until the participant has been recruited into the trial, taking place after all baseline measurements have been done.

Randomization will be performed by the Clinical Research Unit of the Department of Medical Information of the Montpellier University Hospital. Randomization will be stratified by study center. It will be centralized, accessible online, and programmed using the data Capture Software System (EnnovClinical randomization module) according to a minimization algorithm with a 1:1 ratio. The trial manager will be responsible for setting up and testing the randomization process, incorporating stratification by study center using random blocks 1:1, assuring that study groups of approximately the same size will be produced.

The patient number will be automatically generated by EnnovClinical software.

### Blinding

The psychologists performing inclusion, 3-month, and 9-month assessments will be blind to the allocations of the patients to either study arms of the randomized controlled study (RCT). Patients and psychologists performing the ENERGY therapy will be asked to avoid telling the assessor to which group they/the patient belongs to. A psychologist, blind to the randomization process, will perform the assessments, and another psychologist will perform the therapy interventions. Participants will be asked to not reveal their group allocation to the assessors. The data analysts that will be performing the analyses at the conclusion of the trial will also be blinded as to what group the data corresponds to. Unblinding should occur only in exceptional circumstances in case of any adverse events (AE) which will be reported immediately to the investigator.

## Methods: data collection, management, and analysis

### Data collection

Several scales and tests used in this study are comprised within the standardized evaluations performed in the schizophrenia expert centers and are therefore already used on a daily basis in the centers implicated in the study. Each year, all the members of the centers attend quotation sessions in order to ensure inter-rater validity.

For the scales specific to this study (MFI, CAINS, IMI-SR, IHSS, BQ, SCI, IPAQ), we will organize training sessions before the beginning of the protocol in the French expert schizophrenia Network. In addition, all schizophrenia expert centers meet monthly in Créteil, in a meeting organized by the FondaMental Foundation and have specific expertise in clinical, neuropsychological, and biological evaluation in schizophrenia. All teams will be trained in the intervention the year before the beginning of the study to guarantee the homogeneity and quality of treatment across the centers.

Data will be collected or reported in an electronic case report form (e-CRF) developed using the software Capture System EnnovClinical to control for data quality at entry. The connection is via a username and password unique to each specific user and giving access only to the data of the centers’ user. An audit trail function is included allowing a supervision and traceability of all actions from all users.

Concerning biological evaluations, we will have access to specialized platforms for Microbial translocation and inflammatory and immunologic dosing, localized in Nîmes and Montpellier allowing great communication with the study coordinator based in Montpellier.

Once a participant has been enrolled and randomized into their study arm, the study site will put in place every effort to follow the participant throughout the course of the study. In order to improve adherence and prevent loss to follow-up, we will organize phone calls and/or emails that will be sent before the intervention to recall patients of their appointments. Patients will be already following day hospital care, which we believe will also increase adherence to the study. Furthermore, each evaluation will be planned in accordance with each patients’ schedule, and all patients will be phoned 24 h before their evaluation to remind them of their appointment.

If a patient misses their appointment, the psychologist will call him/her. All attempts will be documented in the patient’s medical records. In case of discontinuation, the investigator will perform all examinations scheduled for the final study visit, which includes recording of AEs. In any case, the patient will be treated in accordance with standard care in the center.

Investigators should make every effort to minimize the number of patients lost to follow-up and to obtain a maximum of information on patients lost to follow-up, particularly in the search for any AEs.

### Data management and monitoring

The patients will be identified by the patient code:

- Number of the center (1 digit)
- Number of the patient in the center, by chronological order of selection (2 digits)
- First letter of first name
- First letter of family name

#### Source documents

Source documents are original documents and patient records from which patient data are reported in the e-CRF. The investigator must commit to allow direct access to data sources in the study during inspections or audits.

The source documents that may be used to complete the e-CRF are the patient records. The investigator for each study center commits to allow direct access to the patient records during inspections or audits.

The encrypted data is transmitted to the data-management center via a secure internet connection.

The eCRF will be designed to capture all relevant medical information from patients included in the project. The study methodologist will have a restricted reader-only access to all data in order to monitor the progress and the quality of the data.

The purpose of an audit is to confirm that the study is conducted as per protocol, International Conference on Harmonisation-Good Clinical Practice (ICH-GCP), and applicable regulatory requirements; that the well-being and the rights of the subjects enrolled have been protected; and that the data relevant for the evaluation of the investigational product have been recorded, processed, and reported in compliance with the planned arrangements. The investigators will permit a direct access to all study documents, drug accountability records, medical records, and source data.

#### Data processing

Individual data needed for the study analysis must:

- Be entered in the e-CRF as they are obtained, for both clinical and paraclinical data
- Be anonymized by the investigator
- Be authentified by an electronic signature of the investigator
- All be entered, and missing data must be justified

#### Site training and monitoring procedures

The monitor must present the protocol and all procedures related to the study during an initiation visit performed before the first patient is included. A case report form of completion guidelines will be provided to the investigator.

The monitor will be allowed to have access to all source documents needed to verify the entries on the eCRF and other protocol-related documents.

To ensure accurate, complete, and reliable data, the sponsor or its representatives will do the following:

- Provide instructional material to the study sites, as appropriate
- Provide a start-up training session to instruct the investigator(s) and study coordinator(s). This session will give instruction on the protocol, the prompt and full completion of the clinical report forms, study procedures, and the transmission of data in a timely manner to the clinical database for statistical analyses
- Make periodic visits to the study site
- Be available for consultation and stay in contact with the study site personnel by mail, telephone, and/or fax
- Review and evaluate case report form data and use
- Conduct quality review of database

Routine monitoring visits will be made by the monitors, designated by the sponsor to check compliance with the protocol, the completeness, accuracy, and consistency of the data and adherence to Good Clinical Practice (GCP).

The principal investigator must ensure that eCRFs are completed in a timely manner and must allow periodical access to eCRFs, patient records, drug logs, and all other study-related documents and materials.

The investigator will agree to provide the monitor direct access to the subjects’ source data, which may exist in the form of hospital records, patient files and notes, and laboratory assessment reports and results.

Considering the low risks in this study, there will be no formal Data Safety and Monitoring Board for this study.

### Trial governance and monitoring

The sponsor is responsible for the administrative and financial management of the study, for its quality, and for the choice of the investigators and sites. The sponsor is also responsible in the assessment of sAE, in reporting any suspected unexpected serious adverse reaction (SUSAR) and new events according to the legislation and good clinical practices, to the competent authorities. In addition to the expedited reporting, the sponsor shall submit, once a year, throughout the clinical trial or on request, a safety report to the competent authorities and the Ethics Committee (EC) of the concerned Member States taking into account all new available safety information received during the reporting period. The aim of the annual safety report is to assess the safety conditions of subjects included in the concerned trial(s). It should be the same for the competent authorities (CA) concerned and the Ethics Committee concerned. The sponsor is responsible for the ongoing safety evaluation of the Investigational Product. The sponsor should promptly notify all concerned investigators and the regulatory authorities of findings that could affect adversely the safety of subjects, impact the conduct of the trial, or alter the CA approval/EC’s favorable opinion to continue the trial.

The investigator is responsible for the study quality, the respect of the GCP, and protocol during the conduct of the study. The investigator will ensure information on the study is given to participants and will check that they have given their informed consent. The study coordinator (psychologist in the department of the CHU Adult Psychiatry of Montpellier) will be in charge of coordinating the 9 centers: general organization (follow-up of inclusions of all centers), discussing progress, and study logistics. Each site will have a co-investigator responsible in contacting an appropriate pool of potential subjects and inviting them to participate in the study as well as ensuring the participants’ eligibility. A core project management team consisting of the principal investigator, all co-investigators, and study coordinator will meet monthly to oversee the general running of the trial.

Psychologists in each center will be in charge of assisting the investigators on site for the following:

- Management of study on site
- Reporting of data in the eCRF
- Verification of respect of study procedures
- Recruitments

Another psychologist, from each center, will deliver the ENERGY therapy.

The physicians will be responsible in presenting the study (information, consent, inclusion) and following the patients during the entire study. Study nurses will be responsible in the management of blood sample, freezing, and shipment.

The methodologist will be in charge of the quality of the data and the statistics.

The laboratories implicated in the study will be in charge of the reception, storage, and analysis of blood samples.

GCP requires that the clinical protocol, any protocol amendments, the informed consent and all other forms of patient information related to the study (e.g., advertisements used to recruit patients), and any other necessary documents be reviewed by an Independent Ethics Committee (IEC)/Institutional Review Board (IRB). IEC/IRB approval of the protocol, informed consent and patient information, and/or advertising, as relevant, will be obtained prior to the authorization of enrolling patients in the study. The study shall be conducted as described in this approved protocol. Any revisions/amendments to the protocol will not be permitted without prior approval by the study steering committee. Any amendments to the protocol will require IEC/IRB approval prior to implementation of any changes made to the study design.

### Statistical analysis

The statistical analysis plan (SAP), covering all the analyses to be performed on all data, will be written before database lock for final extraction.

For all collected variables, descriptive statistics will be shown for both study arms in order to verify the initial comparability of the groups. For metric variables, a check as to whether the data can be assumed to be normally distributed will be first conducted. For normally distributed variables, means and standard deviations will be calculated, whereas for skewed variables, medians and ranges will be given. For categorical variables group proportions and contingency tables will be prepared.

All tests of efficacy will be conducted on an intentions to treat (ITT) basis using, in case of missing data, the Last Observation Carried Forward method, and/or multiple or mean imputation [50]. A per protocol sensitivity analysis will also be performed taking into account only patients who completed the whole study without any major protocol deviations. This will enable us to reinforce the stability of results obtained by ITT approach.

Significance level will be set at *p* < 0.05. All statistical analyses will be performed using SAS software (SAS Institute, Cary, NC, USA).

#### Main endpoint analysis

The efficacy of the ENERGY intervention will be studied firstly by comparing the change in fatigue score (using the scores on the MFI) between inclusion/randomization and the 9-month follow-up (primary outcome) in the intervention group compared to the TAU group.

For each subject, the relative difference (delta) between the inclusion and 9-month fatigue score will be calculated. The normality of the delta score will be tested using the Shapiro Wilk’s test. If the distribution is skewed a transformation will be performed to normalize it.

Study arms will be compared between inclusion scores and the 9-month scores using mean differences. These comparisons will be done using Student’s *T* test and general linear regression models adjusting for covariates. Socio-demographic and clinical variables will be tested as potential confounders using general linear regression models, with the *p* value set at 0.10.

A second analysis will be carried out with the general fatigue score as the outcome variable, adjusting for general fatigue score at inclusion.

#### Secondary endpoint analysis

The secondary outcome analyses will be assessed similarly to the main endpoint analyses. Specifically, for normally distributed variables, means and standard deviations will be used to conduct the analyses, whereas for skewed variables, medians and ranges will be used.

- A secondary outcome variable will be constructed, measuring the change in fatigue score (based on MFI scores) between inclusion scores and the 3-month follow-up, representing the end of the ENERGY intervention (for the intervention study arm). Change in fatigue score over the three time points using mean differences in scores between the intervention and TAU study arms will be compared using a mixed-effects model, taking into account the repeated and reversible time-dependent outcome measures.
- Assessment of the effect of the intervention on a range of exploratory outcomes (clinical and cognitive factors, biological markers) measured at the 9-month follow-up will be carried out using either linear regression models or logistic regression models, depending on the type of variable (continuous or binary). These scores will be compared to the initial scores obtained at inclusion. For each outcome variable, the model will be adjusted for the baseline value. These secondary analyses will include measures of:
  - ◦ General symptomatology—scores of the PANSS
  - ◦ Severity in negative symptoms—scores of the CAINS
  - ◦ Depression—scores of the CDSS
  - ◦ Risk of sleep apnea—scores on BQ
  - ◦ Hypersomnolence—scores on IHSS
  - ◦ Insomnia—scores on SCI
  - ◦ Level of physical activity—scores on IPAQ
  - ◦ Quality of life—scores on S-QoL
  - ◦ Functional remission—scores on FROGS
  - ◦ Level of intrinsic motivation—scores on IMI-SR
  - ◦ Intellectual ability—scores on fNART
  - ◦ Learning and recall—scores on CVLT
  - ◦ Attention—scores on TMT
  - ◦ Weight, BMI, abdominal perimeter, hearth rate, blood pressure
  - ◦ Number of steps and distance covered over a week using the pedometer
- A sub-group analysis will be performed on the intervention arm of the RCT, to identify potential factors that are linked to efficacy of the intervention. This main outcome variable will be the change in fatigue score between inclusion and the 9-month follow-up. Linear regression models will be constructed to study the effect of each dependent variable, individually and altogether in a multi-adjusted model, on the outcome.
- Additionally, a cross-sectional analysis of baseline data will be performed to study the biological (inflammatory markers, microbiota) and psychological factors associated with fatigue severity, overall and on each of the five MFI sub-scales, using general linear regression models.

Modifications to the initial SAP will require an amendment to the protocol. Exploratory statistical analyses not documented in the initial SAP will be discussed by the co-investigators and statisticians, in order to establish their importance and acceptability with respect to the study’s main and secondary objectives.

## Discussion

Schizophrenia is a debilitating condition with a severe impact on quality of life, high rates of relapse, and low employment rates, between 10 and 20% in Europe. The total cost in France in 2005 amounted to €3795 million. Fatigue is highly prevalent in schizophrenia and constitutes a significant barrier to employment, social interactions, and quality of life and is likely to have negative impacts on medical comorbidities by constituting an important barrier to physical activity.

However, and surprisingly, certainly because of the overlap between depression and negative symptoms, fatigue is considered to be resistant to psychological and pharmacological interventions and is a neglected phenomenon in clinical care of individuals with schizophrenia. Interventions for fatigue have been shown to be effective for numerous other medical conditions with or without cognitive deficits but have never been studied in schizophrenia.

Our project is to propose the first randomized controlled study designed to alleviate fatigue severity in schizophrenia, which could lead to direct short-term improvements of quality of life, affective state, autonomy, and motivation and at long-term reduction of cardiovascular risk factors and morbidity. In addition, the expected benefit for patients is to produce an integrative model, taking into account psychological and biological factors, in order to better understand and support fatigue in schizophrenia.

### Ethical considerations and confidentiality

There are to our knowledge no potential risks involved with participating in the intervention proposed in this study other than the usual risk linked to moderate walking.

Risks concerning the collection of blood samples were weak: unease, bruise, or anxiety.

Risks concerning neuropsychological and clinical evaluations are weak: anxiety, tension, and tiredness.

Regarding the vigilance of the project which are responsibilities of the investigator and sponsor, the reporting of serious adverse events (sAE) and annual safety reports will be monitored and carried out in accordance with regulations.

#### Collection of AEs

AEs will be collected non-systematically through spontaneous report by participants or other members of the team. Complete and appropriate data on all AEs experienced during the clinical trial will be recorded on the AE form of the CRF on an ongoing basis for the duration of the study. Each AE report shall include a description of the event, an assessment of its seriousness, its duration, intensity, relationship to the study medication, other causality factors (if any), any concomitant medication dispensed, actions taken with the study drug or other therapeutic interventions, and outcome at the end of the observation period. For each AE, a separate AE form will be filled out.

All AEs (whether expected or unexpected) will be recorded in the eCRF and will be reported in the subsequent trial publications except for the following:

- Admission for social or administrative reasons
- Hospitalization predefined by the protocol
- Hospitalization for scheduled medical or surgical treatment prior to research

AEs will be followed up until their resolution or stabilization. However, the observation period will be cut off after the last patient has finished his final visit (LPLV).

#### Notification of SAEs

All SAEs occurring during the study must be reported on the SAE form to the Pharmacovigilance Clinical Trial Department of CHU Montpellier handling pharmacovigilance matters in this study on behalf of the sponsor.

The Pharmacovigilance Clinical Trial Department of the sponsor must be informed immediately (i.e., within 24 h) of the occurrence of any SAE by fax or by e-mail.

The investigator is required to include any investigations as may be indicated to elucidate the nature or the causality of the SAE. This may include additional lab tests, histopathological examinations, and consultations with other healthcare professional or if the patient dies, any post mortem findings. The relation between the event and the study drugs should be evaluated by the investigator.

After the initial report, the investigator is required to follow each patient until the end of the SAE and to provide further information on the patient’s condition to the sponsor. The investigator must also record all SAEs in the CRF.

Some serious adverse events that do not require immediate reporting will only be documented on the CRF/source data such as:

- Serious adverse events occurring after given informed consents, but before starting study procedures/application of study medication
- Hospitalizations

### Trial withdrawal or discontinuation

Study participation is voluntary. The subjects have the right to withdraw their consent from the study at any time and for any reason, without jeopardizing their medical care. The investigator also has the right to withdraw patients in agreement with the sponsor or his delegate. The investigator has the right to withdraw subjects from the trial in the event of concurrent illness, adverse events, and treatment failure after a prescribed procedure, protocol violations, and other reasons.

Subjects must be withdrawn from the study under the following circumstances:

- The subject withdraws consent.
- Development of another illness or condition which would interfere with the subject’s continued participation.
- Discovery that the subject entered the clinical trial in violation of the protocol or occurrence of a significant protocol violation during the clinical trial.
- Any situation that in the opinion of the investigator would pose inacceptable risks to the subject if trial participation is continued.

For any discontinuation, the investigator should obtain all required details and should document the date and reason of the premature termination in the CRF.

If the reason for withdrawal is an AE, the specific event will be recorded in the CRF. The investigator will make thorough efforts to document the outcome.

Patients prematurely withdrawn or lost to follow-up will be analyzed at least in the safety set and in the conditions of confidentiality.

### Definition of the end of trial

The end of the study is the date of the last visit of the last subject undergoing the intervention.

#### Stopping rules

The sponsor may discontinue the study at any time for any of the following reasons:

- Insufficient rate of recruitment
- Serious unsolvable problems with data quality
- Occurrence of unpredictable conditions at the trial site(s) demanding the discontinuation of the clinical trial
- Upcoming scientific findings or ethical concerns during the conduct of the clinical trial demanding the discontinuation

The reasons for discontinuation of the clinical trial should be precisely documented.

If an investigator has ethical concerns about the conduct of the clinical trial, the coordinating investigator has to be informed without delay.

In case of premature discontinuation of the clinical trial by the sponsor or the coordinating investigator, the responsible national competent authorities and ethic committees must be informed within the timelines required by European and national regulations.

The Competent Authorities may suspend or prohibit a study if it considers that the conditions of authorization are not being met or has doubt about the safety or scientific validity of the study. In case of a SUSAR, the investigator will inform the sponsor for stopping the trial and for informing regulatory agencies.

Other than the annual safety evaluations, mentioned above, we do not plan to carry out any interim analyses.

## Supplementary information

**Additional file 1.** SPIRIT 2013 Checklist: Recommended items to address in a clinical trial protocol and related documents.

## Data Availability

Not applicable.

## Abbreviations

AE: Adverse events
BHQ1: Black Hole Quencher 1
BQ: Berlin Questionnaire
BMI: Body mass index
CAINS: Clinical Assessment Interview for Negative symptoms
CBT: Cognitive behavioral therapy
CDSS: Calgary Depression Scale for Schizophrenia
CD14: Cluster of differentiation 14
CHU: Centre Hospitalier Universitaire; University Hospital
CONSORT: Consolidated Standards of Reporting Trials
CRF: Case report form
CRP: C-reactive protein
CVLT: California Verbal Learning Test
DNA: Deoxyribonucleic acid
DSM-5: Diagnostic and Statistical Manual of Mental Disorders, fifth edition
EC: Ethics Committee
eCRF: Electronic case report form
ENERGY: Acronym for the fatigue management intervention in this study
EDTA: Ethylenediaminetetraacetic acid
FAM: Fluorescein amidites
FABP: Fatty acid-binding proteins
fNART: French National Adult Reading Test
FROGS: Functional Remission of General Schizophrenia
GM-CSF: Granulocyte-macrophage colony-stimulating factor
GPS: Global Positioning System
HRQL: Health-related quality of life
ICH-GCP: International Conference on Harmonisation-Good Clinical Practice
IEC: Independent Ethics Committee
I-FABP: Intestinal fatty acid binding protein
IIHSS: Idiopathic Hypersomnia Severity Scale
IL: Interleukins
IMI-SR: Intrinsic Motivation Inventory for Schizophrenia Research
IRB: Institutional Review Board data
ITT: Intention to treat
LBP: Lipopolysaccharide binding protein
LDCR: Long Delay Cued Recall
LDFR: Long Delay Free Recall
MFI: Multidimensional Fatigue Inventory
IPAQ: International Physical Activity Questionnaire
IFN: Interferons
OSA: Obstructive sleep apnea
PANSS: Positive And Negative Syndrome Scale
PCR: Polymerase chain reaction
RCT: Randomized controlled study
SAE: Serious adverse events
SAS: Statistical analysis plan
SCI: Sleep Condition Indicator
SDCR: Short Delay Cued Recall
SDFR: Short Delay Free Recall
S-QOL: Schizophrenia Quality of Life Questionnaire
SUSAR: Suspected unexpected serious adverse reaction
SZ: Schizophrenia
SD: Standard deviation
TAU: Treatment as usual
TMT: Trail Making Test
TNF: Tumor necrosis factors
